# Circularly Polarized Photodetectors Based on Chiral Materials: A Review

**DOI:** 10.3389/fchem.2021.711488

**Published:** 2021-09-08

**Authors:** Can Zhang, Xiaohong Wang, Longzhen Qiu

**Affiliations:** ^1^ National Engineering Lab of Special Display Technology, State Key Lab of Advanced Display Technology, Academy of Opto-Electronic Technology, Hefei University of Technology, Hefei, China; ^2^ Anhui Key Laboratory of Advanced Functional Materials and Devices, School of Chemistry and Chemical Engineering, Hefei University of Technology, Hefei, China; ^3^ Key Laboratory of Measuring Theory and Precision Instrument, Hefei University of Technology, Hefei, China

**Keywords:** circularly polarized light, photodetector, chiral materials, circularly polarized photodetectors, direct detection

## Abstract

Circularly polarized light (CPL) plays an important role in many photonic techniques, including tomographic scanning based on circular polarization ellipsometry, optical communication and information of spin, and quantum-based optical calculation and information processing. To fully exploit the functions of CPL in these fields, integrated photoelectric sensors capable of detecting CPL are essential. Photodetectors based on chiral materials can directly detect CPL due to their intrinsic optical activity, without the need to be coupled with polarizers and quarter-wave plates as in conventional photodetectors. This review summarizes the recent research progress in CPL photodetectors based on chiral materials. We first briefly introduce the CPL photodetectors based on different types of chiral materials and their working principles. Finally, current challenges and future opportunities in the development of CPL photodetectors are prospected.

## Introduction

**FIGURE 1:**
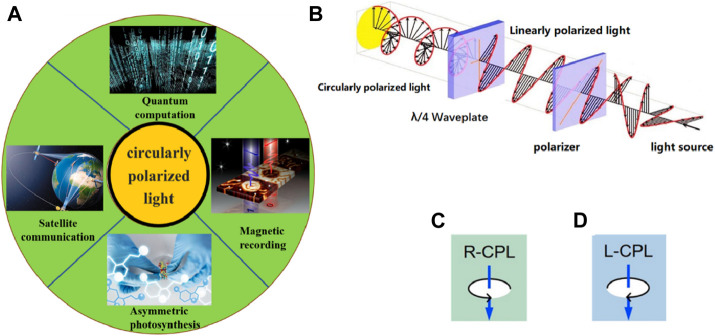
**(A)** Various applications of CPL, such as quantum computations, satellite communication, asymmetric synthesis, and magnetic recording. **(B)** Schematic diagram of the experimental setup to generate CPL. According to the rotation direction of the light vector, circularly polarized light is divided into **(C)** Right-handed CPL and **(D)** Left-handed CPL.

As one of the inherent and important properties of light, polarization provides information beyond light intensity and spectra and can be applied in remote sensing, target identification, astronomical detection, and medical diagnosis. In particular, circularly polarized light (CPL) can be obtained by superimposing two linearly polarized light waves with equal frequencies and whose vibration direction is perpendicular to each other. CPL is similar to the natural light, but the light vector of CPL varies regularly, whereas the vector of natural light varies irregularly. Due to its rich optical information and lack of angle dependence, CPL is widely used in various optical technologies and devices, including quantum computing (Ganichev and Prettl, 2003), satellite communication (Sherson et al., 2006), magnetic recording (Nordsieck et al., 2003; Pernechele et al., 2003), and asymmetric synthesis (Plum and Zheludev, 2015; Chen et al., 2016) (Figure 1A). In the laboratory, CPL is generated by passing a beam of natural light through a polarizer and quarter-wave plate, as shown in Figure 1B. The electric field vector travels along a helical trajectory, either clockwise or counterclockwise. According to the rotation direction of the light vector, circularly polarized light can be divided into left-handed and right-handed circularly polarized light (LCPL and RCPL), as shown in Figures 1C,D. Left-handed and right-handed CPL can be used as two independent channels to transmit information, thus doubling the transmission speed compared with unpolarized light (Etienne Rochat and Parker, 2004). The traditional method of detecting circularly polarized light (CPL) is as follows: CPL is changed to linearly polarized light by passing it through a quarter-wave plate and then passing the linearly polarized light through a polarizer with a known direction of polarization, and finally, the light intensity of the emitted light is detected using an ordinary photodetector. In this way, CPL is detected. However, this method of detection involves multiple optical components, making it challenging to realize miniature and integrated CPL detectors.

CPL photodetectors based on chiral materials can distinguish between LCPL and RCPL depending on the different output signals under the illumination of LCP and RCP. Since inorganic semiconductors (e.g., silicon and III–V semiconductors) do not have intrinsic chirality, common photodetectors cannot detect the polarization state of CPL directly and must be coupled with quarter-wave plates and linear polarizers (Sherson et al., 2006; Yu et al., 2012; Turner et al., 2013). The multiple optical elements and complex processing methods hinder the miniaturization and integration of circularly polarized photodetectors. Thus, it is important to develop a miniaturized and integrated device for directly detecting CPL.

Chiral materials are defined as objects that cannot be superimposed with their mirror image. The unique properties of chiral materials have important applications in various fields, such as medicine, biology, and quantum technology (Gisin and Thew, 2007; Humphreys et al., 2018; Liao et al., 2018). In particular, chiral materials exhibit differential absorption of LCP and RCP, i.e., circular dichroism (CD). Therefore, chiral materials provide an opportunity to fabricate photodetectors for the direct detection of CPL. Unlike common photodetectors, photodetectors based on chiral materials do not require additional optics to detect CPL. According to the different components of the active layer, CPL-sensitive photodetectors can be classified into organic semiconductors, inorganic semiconductors, and organic–inorganic hybrid perovskite–based circularly polarized photodetectors.

In this review, we focus on the recent progress of CPL photodetectors based on chiral materials. The working principle and classification of CPL photodetectors are introduced, and then we summarize the remaining problems of reported CPL photodetectors and propose several strategies to improve their performance. Furthermore, we look forward to the potential applications of CPL photodetectors in the future, including wearable electronics, optical imaging, and secure communication. Finally, a summary and outlook on the development of CPL photodetectors is given.

## Device Structure and Operating Mechanisms of Circularly Polarized Light Photodetectors

### Device Structure

**FIGURE 2:**
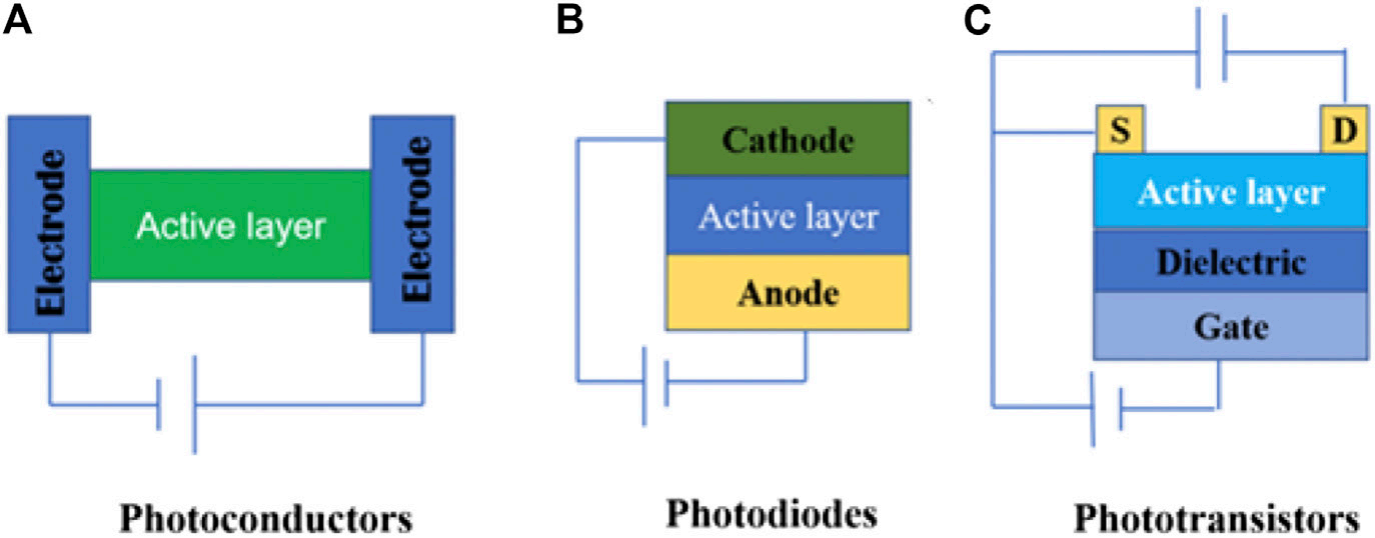
Typical device configuration of CPL-sensitive photodetectors. **(A)** Photoconductors. **(B)** Photodiodes. **(C)** The device configuration of phototransistor. S and D represent the source and drain electrodes of the phototransistors, respectively.

According to different device architectures, CPL-sensitive photodetectors can be categorized into photoconductors, photodiodes, and phototransistors, as shown in Figure 2. Among these, photoconductors and photodiodes are two-terminal devices, while phototransistors are three-terminal devices that have three electrodes: source, drain, and gate.

### Operating Mechanisms of Circularly Polarized Light Photodetectors

#### Operating Mechanisms of Photoconductors

The typical structure of a photoconductor includes an organic semiconductor layer contacted by two metal electrodes with ohmic contacts, as shown in Figure 2A. In the dark, photoconductors exhibit large resistance because of a low carrier concentration in the organic semiconductor. Under the illumination of CPL, photo-induced carriers are generated in the organic semiconductor layer, and they become more conductive under suitable illumination. In photoconductors, one type of charge carrier is recirculated between the two symmetrical electrodes until they recombine with oppositely charged carriers. They usually show high photoconductivity gain and high responsivity due to multiple carrier recirculation.

#### Operating Mechanisms of Photodiodes

Photodiodes are two-terminal configurations, which are very similar to those of photoconductors (Figure 2B), but their working mechanisms are quite different from those of photoconductors. The working mechanism of photodiodes is as follows: First, the semiconductor layer generates excitons (bound electron–hole pairs) under CPL illumination. Then, excitons are separated into photogenerated carriers assisted by the built-in potential or an applied voltage, and the directional movement of carriers under the effect of an external electric field generates a current. Finally, the free charges are collected by external electrodes to generate photocurrent. Compared to CPL photoconductors, CPL photodiodes typically show lower dark currents and faster response speed (Zhou et al., 2009).

#### Operating Mechanisms of Phototransistors

Phototransistors are typical three-terminal configurations of organic field-effect transistors, as shown in Figure 2C. The three electrodes refer to the source, drain, and gate. In the dark, the channel resistance between the source and drain electrodes of the phototransistors is modulated by the gate electrode. Under the illumination of CPL, the channel resistance of the phototransistors can be modulated by the incident light. Illumination increases the carrier concentration in the channel, thereby increasing the output current and converting the optical signal into an electrical signal. CPL phototransistors have both the signal amplification function of transistors and polarized light detection ability. In addition, compared with CPL photodiodes, CPL phototransistors usually show higher responsivity and lower noise (Konstantatos et al., 2012; Yu et al., 2013; Wang et al., 2017; Ji et al., 2019).

#### Direct Detection of Circularly Polarized Light Through the Device

The details of detection of circularly polarized light through devices are as follows: 1) The circular polarized light is energetic irrespective of the polarization state, when it irradiates the active layer of the device. The active layer absorbs the photon energy and generates the photogenerated carriers, which flow to the electrode under the action of external electric field and generate photocurrent. The output current of the device when irradiated by CPL is greater than the current of the device in the dark state; hence, it can detect CPL. 2) The chiral active layer absorbs the left- and right-handed CPL differently. Therefore, the numbers of photogenerated carriers generated in the active layer are different when the left- or right-handed CPL is irradiated. The value of photogenerated current is also different, and the two polarization states of circularly polarized light can be distinguished as per the difference in the value of output current of the device (Yang et al., 2013a; Shang et al., 2017; Cheng et al., 2020a).

## Performance Metrics for Circularly Polarized Light Photodetectors

There are several key parameters used to evaluate the performance of CPL photodetectors, including the spectral range, photosensitivity (*P*), responsivity (*R*), specific detectivity (D*), the anisotropy factor of responsivity (*g*_*res*_, which describes the ability to distinguish LCPL and RCPL), external quantum efficiency (*EQE*), and response speed, which are summarized as follows (Li et al., 2019).

1) The spectral range of CPL responsivity: Each CPL photodetector only responds to a specific wavelength range.
2) Photosensitivity (*P*) measures the increase in signal upon illumination and is defined as

$$P=\frac{I_{light}-I_{dark}}{I_{dark}},$$ (1)

where *I*_*light*_ and *I*_*dark*_ denote the current under illumination and in the dark, respectively.

3) Responsivity (*R*) quantifies the ability of a CPL detector to transform light into an electric current, whose value is defined as follows:

$$R=\frac{I_{light}-I_{dark}}{E_{light}},$$ (2)

where *E*_*light*_ denotes the power of CPL.

4) Specific detectivity (D*) is calculated using the following equation (Liu et al., 2014; Shao et al., 2015):

D∗=(SB)12NEP, (3)

NEP=in2−12R, (4)

where *B* denotes the bandwidth, *NEP* denotes the noise equivalent power, and *i*_*n*_^*2-1/2*^ refers to the RMS value of the noise current.

5) The anisotropy factor of responsivity *g*_*res*_ is defined as

$$g_{res}=\frac{2\left(R_{L}-R_{R}\right)}{R_{L}+R_{R}},$$ (5)

where *R*_*L*_ and *R*_*R*_ denote the responsivities of CPL detectors under the illumination of LCPL and RCPL, respectively.

6) External quantum efficiency (*EQE*) is defined as the ratio of the number of photogenerated carriers that practically enhance the drain current to the number of photons irradiated onto the device (Yu et al., 2013), whose value can be calculated by the following formula:

$$EQE=\frac{hcR}{e\lambda },$$ (6)

where *h* is the Planck constant, *c* is the speed of light, *R* is the responsivity, *e* is the fundamental unit of charge, and λ is the maximum absorption wavelength.

7) Linear dynamic range (*LDR*) indicates whether the photocurrent is linearly proportional to the incident optical power within a certain range, whose value is defined using the following formula:

$$LDR=20\;\text{log}\;\left(\frac{Imax}{Imin}\right),$$ (7)

where *I(max)* and *I(min)* denote the maximum and minimum current, respectively.

8) The response speed of a photodetector is characterized as the rise time (t_rise_) and fall time (t_decay_) of a current signal under the stimulation of an optical signal. The rise times are defined as the time required for the current to rise to 90% of its maximum value under the illumination of incident light. The decay time is defined as the time required for the current to decay to 10% of its minimum value after removing incident light (Chen et al., 2020).

## Materials for Circularly Polarized Photodetectors

### Chiral Organic Semiconductors

Organic semiconductors are very attractive for light detection applications due to their intrinsic advantages such as large absorption coefficients, good solution processability, and lightweight nature (El Gemayel et al., 2012; Yu et al., 2013; Zhou et al., 2018; Li et al., 2019). Moreover, the optical energy gap and the hole and electron transport properties of organic semiconductors can be easily tuned through chemical synthesis, allowing them to achieve spectrally selective detection ranges from UV (Zhou et al., 2009; Li et al., 2020) to visible (Bristow et al., 2020), and to near-IR wavelengths (Yang et al., 2008; Xu et al., 2013; Wang et al., 2016; Ma et al., 2018; Simone et al., 2018; Li et al., 2019; Jang et al., 2020). Chiral organic semiconductors have the impressive electrical properties of organic semiconductors, as well as the chiral activity of chiral materials, making them ideal candidates for use as the active layer of CPL photodetectors.

### Chiral Organic Small Molecules and Supramolecules

**FIGURE 3:**
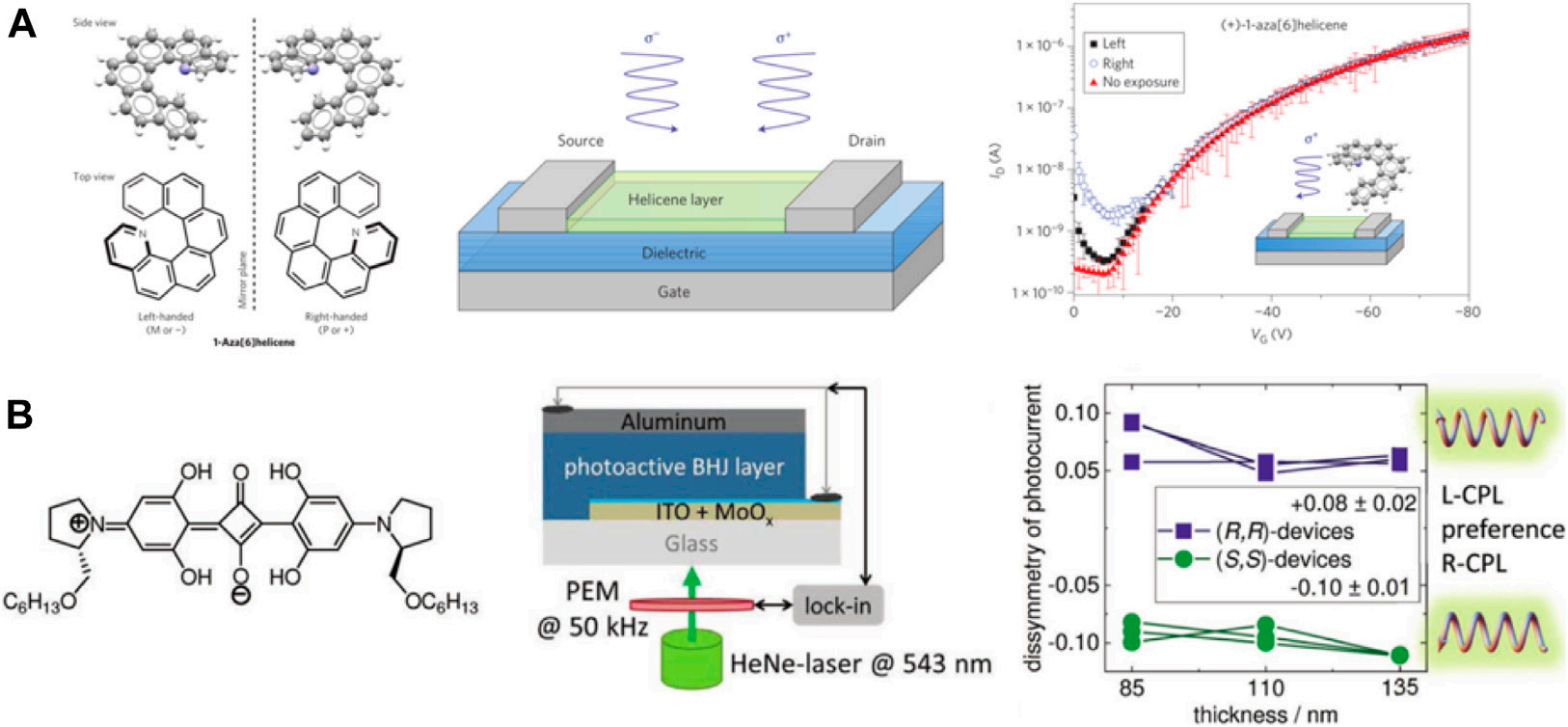
**(A)** Molecular structure of (+)-1-aza[6]helicene, device structure of the CPL phototransistors, and transfer characteristic curve of the phototransistors based on (+)-1-aza[6]helicene in the dark or under CPL illumination (λ = 365 nm, power = 10 mW cm^2^) (Yang et al., 2013b). Reproduced with permission. Copyright 2013, Nature Publishing Group. **(B)** Chemical structure of ProSQ-C6, a schematic diagram of the device architecture, and dissymmetry of short-circuit current g_lsc_ under illumination with 543 nm CPL (Schulz et al., 2019). Reproduced with permission. Copyright 2019, Wiley-VCH.

Organic small molecules are suitable candidates for optoelectronic applications because they can be efficiently purified and can form ordered structures, which contribute to high charge-carrier mobilities (Li et al., 2014; Pan et al., 2019). Among these, the helicenes are unique because their helically chiral architecture and fully conjugated structure give these molecules strong chiroptical properties and moderate charge transport efficiencies. For instance, in 2013, Campbell et al. reported a CPL photodetector based on chiral small molecules (+)-1-aza[6]helicene and (-)-1-aza[6]helicene (Figure 3A). The device showed notably different photocurrents between RCP and LCP illumination and with a responsivity of 10 mA W^−1^ at a wavelength of 365 nm (Yang et al., 2013a). It was the first organic semiconductor–based phototransistor that could selectively detect RCP and LCP. This work opened up the possibility to detect CPL in a highly integrated photon platform, which is beneficial to the preparation of miniaturized and integrated devices for the direct detection of CPL. In addition to the chiral small molecules 1-aza[6]helicenes, chiral squaraine thin films can also be used to construct CPL photodetectors due to the nature of their excitonic coupling (Schulz et al., 2018). In 2019, Schiek et al. demonstrated a self-powered heterojunction photodiode detector based on a mixture of chiral ProSQ-C6 and a conventional fullerene acceptor phenyl-C61-butyric acid methyl ester (PCBM) in a 2:3 mass ratio. The structural formula of chiral ProSQ-C6 and device structure are shown in Figure 3B. The results demonstrate that the current dissymmetry amounted to *g*_*ISC*_ = 0.08 ± 0.02 for the (*R,R*)-devices and to *g*_*ISC*_ = −0.10 ± 0.01 for the (*S,S*)-devices. The values were independent of the layer thickness for the inspected range and have therefore been averaged using all the measurements for the respective enantiomer. Using these results, the overall efficiency for the circular polarization discrimination amounted to about 5% with preferential sensitivity of (*R,R*)-enantiomer toward LCPL and preferential sensitivity of (*S,S*)-enantiomer toward R-CPL (Schulz et al., 2019). To develop high-performance chiral organic semiconducting molecules, a distorted π-system is required for strong coupling with circularly polarized light (CPL), whereas planar π-stacking systems are necessary for high charge-carrier mobility. To address this concern, Zhang et al. introduced a skeleton merging approach through the distortion of a perylene diimide (PDI) core with four fused heteroaromatics to form an ortho-π-extended PDI double-[7]heterohelicene (Zhang et al., 2021). The PDI double helicene inherits high dissymmetry factor from the helicene skeleton. The extended π-planar system concurrently retains a high level of charge transport properties. Also, ortho-π-extension of the PDI skeleton provides near-infrared (NIR) light absorption and ambipolar charge transport abilities, endowing the corresponding organic phototransistors with high photoresponsivity of 450 and 120 mA W ^−1^ in p- and n-type modes, respectively. These characteristics come along with high external quantum efficiency (89%) under NIR light irradiations. This research uses chiral organic semiconductors to achieve high-performance broadband CPL detection up to the NIR spectral region.

**FIGURE 4:**
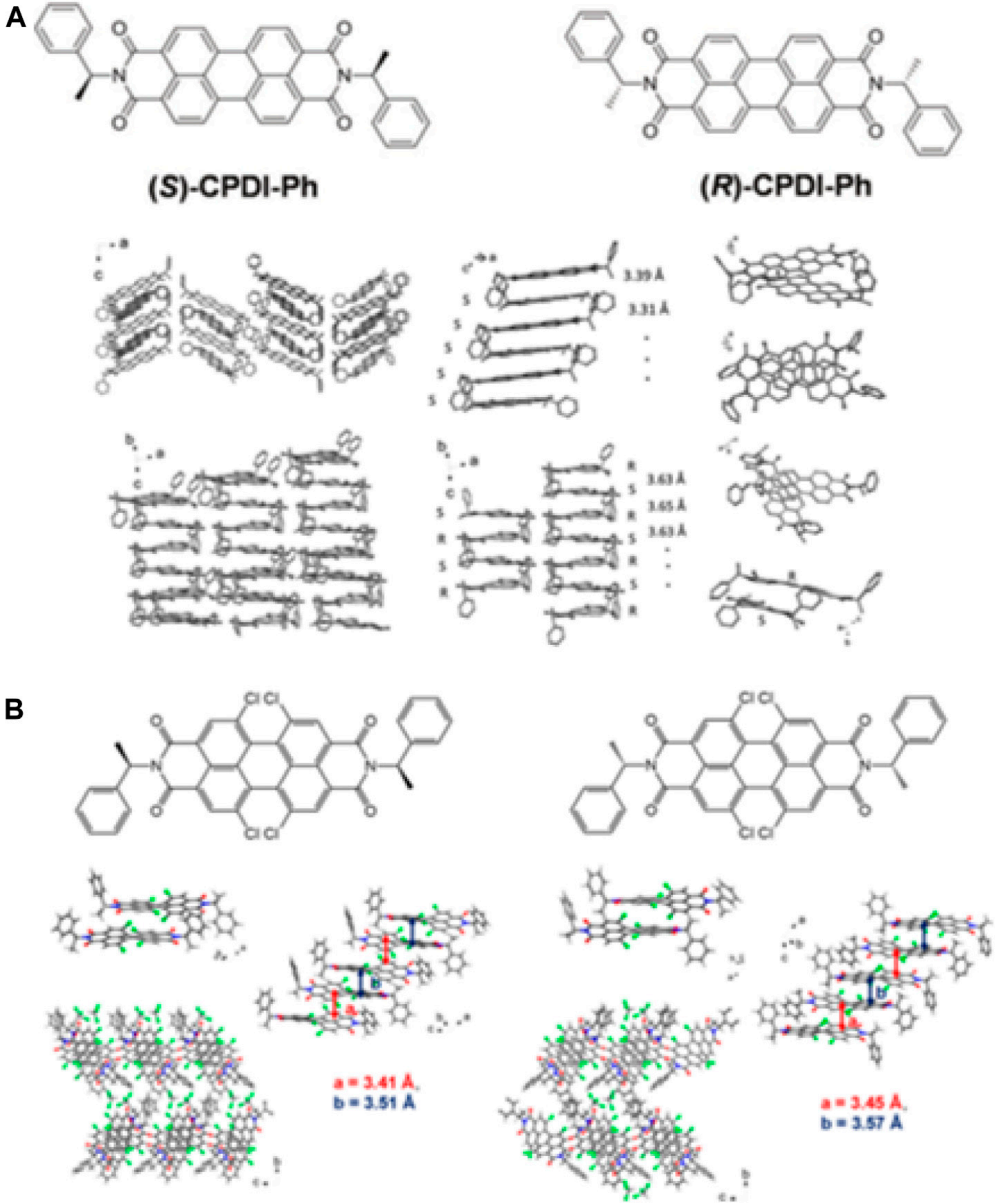
**(A)** Chemical structure of (*S*)-CPDI-Ph and (*R*)-CPDI-Ph and crystal structure of homochiral (*S*)-CPDI-Ph NWs and heterochiral (Rac)-CPDI-Ph NWs (Shang et al., 2017). Reproduced with permission. Copyright 2015, Wiley-VCH. **(B)** Chemical structure of (*R*)-CLCPDI-Ph and (*S*)-CLCPDI-Ph and crystal structure of (*S*)-CLCPDI-Ph-CF and (*R*)-CLCPDI-Ph-CF (Shang et al., 2020). Reproduced with permission. Copyright 2020, American Chemical Society.

In addition to chiral small molecules, chiral supramolecules are asymmetric supramolecular nanostructures that are obtained by the dissymmetrical assembly of conjugated molecules with controlled molecular interactions or the incorporation of conjugated molecules into helical templates (Schulz et al., 2018). Chiral supramolecules can potentially further improve the performance of CPL photodetectors due to their chiral amplification effect. In 2015, Shang et al. reported the synthesis of *N*,*N′*-bis-(1′-phenylethyl)perylene-3,4,9,10-tetracarboxyldiimide (CPDI-Ph) *n*-channel semiconductors with a chiral substituent at the imide position of chiral enantiomers and fabricated their supramolecular nanomaterials via self-assembly, as shown in Figure 4A. Phototransistors based on homochiral nanomaterials show superior charge transport, as well as higher photoresponsivity and dissymmetry factors compared with their thin film counterparts due to well-ordered supramolecular packing. Since single-crystal CPDI-Ph has fewer grain boundaries, single NW-OPTs have achieved maximum *R* and *P* values of 334 A W^−1^ and 7.57 × 10^4^ under 460 nm CPL. In addition, the NW-OPTs reached a maximum *EQE* value of 8.81 × 10^4^% at a gate voltage of 82 V. Importantly, this chiroptical sensing can be realized in the visible region (450 nm), giving it great potential applications in image sensors, optical imaging, and security-enhanced optical communication (Shang et al., 2017). Using the above strategy, their group also synthesized a pair of 5,6,12,13-tetrachloro-2,9-bis(1-phenylethyl)anthra[2,1,9-def:6,5,10-d′e′f′]-diisoquinoline-1,3,8,10(2H,9H)-tetraone (CLCPDI-Ph) enantiomers by introducing four chlorine atoms at the bay position of CPDI-Ph. The obtained CLCPDI-Ph enantiomers self-assembled into quasi-2D single crystals using the synergistic effect between π–π interactions and steric effects, as shown in Figure 4B. The phototransistor based on 2D chiral organic semiconductor crystals (*R*)-C1CPDI-Ph-CF achieved maximum *P*, *R*, D*, and *EQE* values of 5.7 × 10^5^, 187 A W^−1^, 1.3 × 10^15^ Jones, and 5.0 × 10^4^%, respectively. This series of works provided a new strategy for the construction of CPL photodetectors with excellent performance based on chiral supramolecular assemblies (Shang et al., 2020).

### Chiral Conjugated Polymers

**FIGURE 5:**
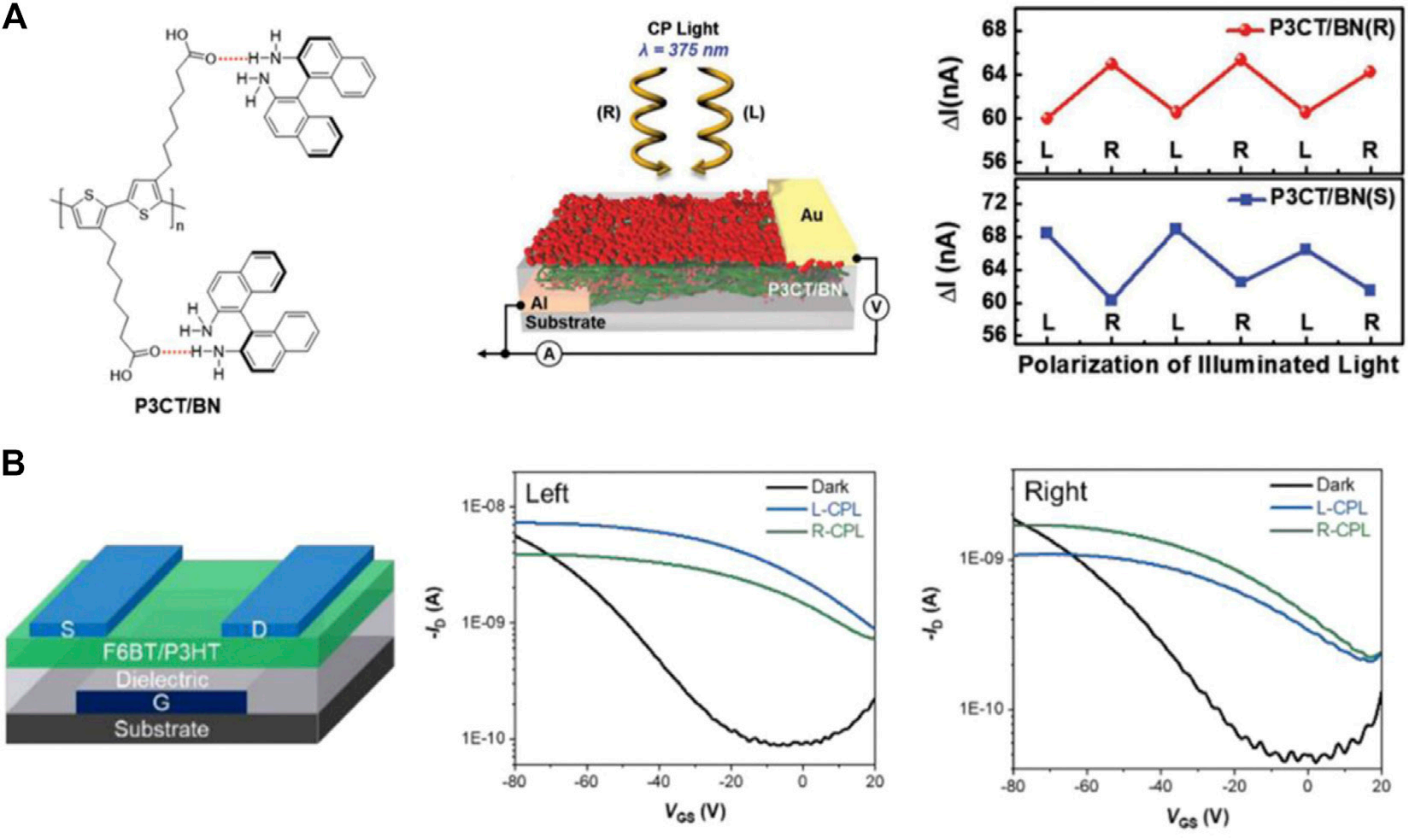
**(A)** Chemical structures of P3CT and BN and a schematic illustration of the hydrogen bonding interactions leading to the formation of a P3CT and BN hybrid system. Device structure of the photodiode based on the P3CT/BN hybrid thin film. Variation in the maximum photocurrent of the photodiodes based on a P3CT/BN (*R*) and P2CT/BN (*S*) hybrid thin film under the illumination of LCPL and RCPL, respectively (Kim et al., 2019). Reproduced with permission. Copyright 2015, Wiley-VCH. **(B)** Schematic structure of the phototransistor based on the F6BT/P3HT blended thin film and transfer curve of the enabled F6BT/P3HT OFETs under the illumination of LCPL and RCPL (Cheng et al., 2020b). Reproduced with permission. Copyright 2020, The Royal Society of Chemistry.

Conjugated polymers are suitable candidates for CPL detection due to their good film-forming properties, solubility, and excellent optoelectronic performance (Yang et al., 2013b; Lee et al., 2017; Wan et al., 2019; Cheng et al., 2020a). Because of their easy solution processing, CPL photodetectors based on chiral polymers are suitable for large-scale fabrication, which is beneficial to the miniaturization and integration of CPL photodetectors. In 2019, Lim et al. obtained chiroptical semiconductor thin films by blending poly[3-(6-carboxyhexyl)thiophene-2,5-diyl] (P3CT) with the chiral molecule 1,1-binaphthyl (BN). The chiroptical activity of P3CT/BN could be amplified by a controlled crystallization and phase separation process (Kim et al., 2019). Then, the photodiode based on P3CT/BN (R) was constructed, as shown in Figure 5A. The device showed a higher photocurrent when exposed to RCPL, than when exposed to LCPL, demonstrating its ability to detect CPL. Moreover, the device based on P3CT/BN heterojunction thin films achieved an average dissymmetry factor of 0.1. In 2020, our group and co-researchers, taking advantage of the chirality induction of achiral fluorene-*alt*-benzothiadiazole–based conjugated polymers, first proposed and experimentally demonstrated a novel achiral organic semiconductor–based direct CPL photodetector, as shown in Figure 5B. The circularly polarized discrimination ability of the organic field-effect transistors could be enabled via external irradiation of the CPL without the use of chiral additives, and the circular polarization sensing of the devices was rigorously controlled by the handedness of inducing light (Cheng et al., 2020b).

### Inorganic Materials

**FIGURE 6:**
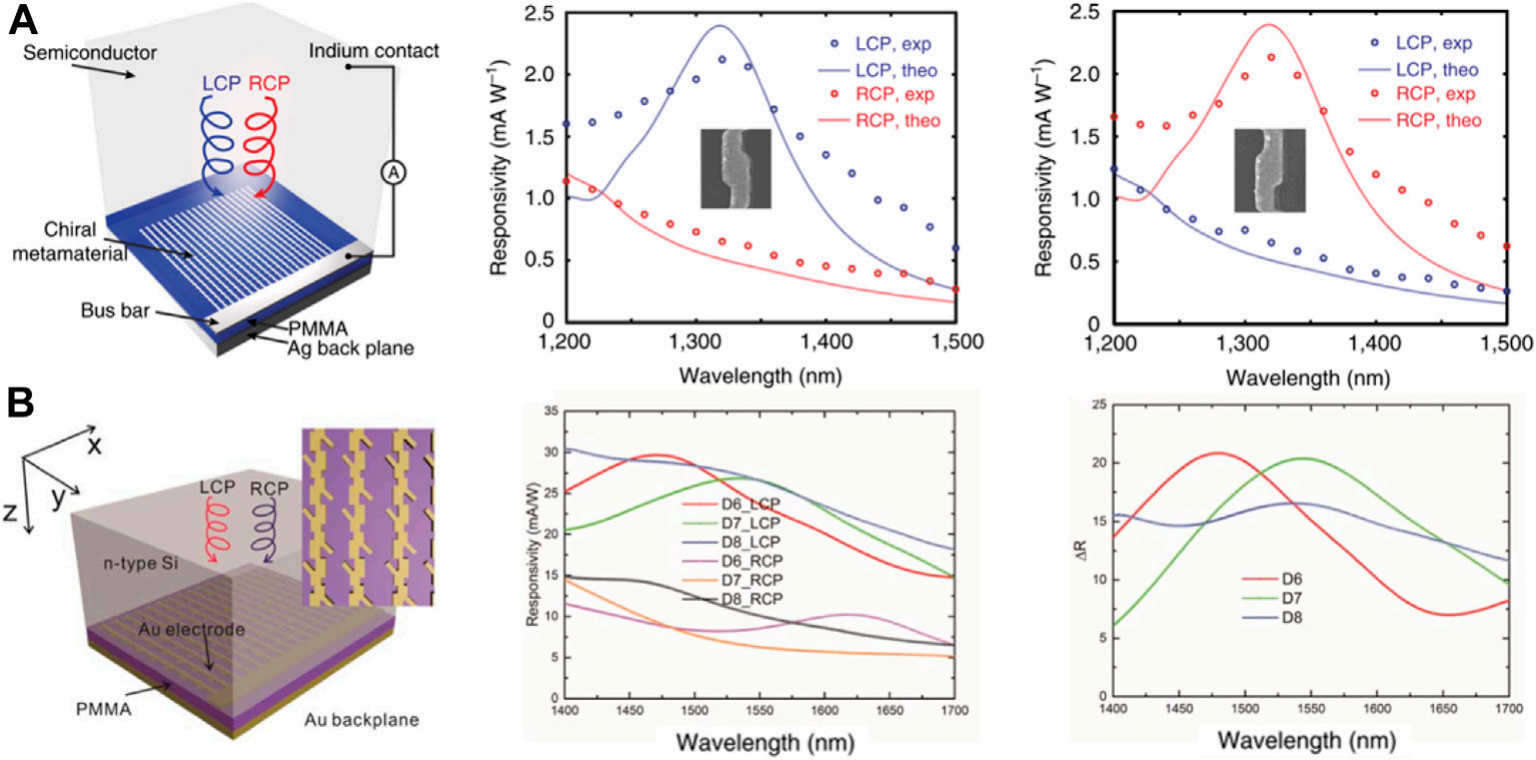
**(A)** Shematic of a CPL detector consisting of a chiral metamaterial integrated with a semiconductor that serves as a hot electron acceptor and experimentally measured optical absorption spectra under LCP and RCP illumination for LH and RH metamaterials (Li et al., 2015). Reproduced with permission. Copyright 2015, Nature Publishing Group. **(B)** Schematic of a 2D CPL photodetector integrated on n-type silicon, responsivity of the CPL photodetector based on 2D chiral metamaterials, and different optical responses of the three devices (Xiao et al., 2019). Reproduced with permission. Copyright 2019, IOP Publishing Ltd.

Compared with organic semiconductors, inorganic semiconductors have stable structures, controllable band structures, and predictable characteristics, which make them suitable for constructing high-performance CPL photodetectors (Teng et al., 2018; Xie et al., 2019; Pak et al., 2020). In 2015, Valentine et al. demonstrated a CPL photodetector based on engineered CD in plasmonic nanostructures and the hot electron–based transfer process (Figure 6A) (Li et al., 2015). The LH metamaterial absorbed LCP light at 1340 nm, while it largely reflected RCP light, and the RH metamaterial showed a nearly opposite response. The device based on this chiral metamaterial achieved a polarization discrimination ratio of 3.4 and a photoresponsivity difference of 1.5 mA W^−1^, demonstrating its ability to detect and distinguish between left- and right-handed CPL in an ultra-compact detector geometry. The two-dimensional (2D) structure is a 2D matrix with an orderly microstructure. Compared with 3D CPL detectors, 2D CPL detectors are easier to fabricate due to their much simpler geometry. The benefit lies in the development of CPL detectors with higher efficiencies. In 2019, Xiao et al. reported a CPL detector based on a 2D embedded chiral nanostructure with a 40 nm-thick center-symmetrical “Y”-shaped gold (Au) antenna, as shown in Figure 6B. Compared with traditional CPL photodetectors, the thin embedded nanostructures achieved a higher internal quantum efficiency (*IQE*) by increasing the emission probability of hot electrons. The final CPL detector based on 2D embedded chiral nanostructures achieved a photoresponsivity difference of 21 mA W^−1^ at 1550 nm (Xiao et al., 2019).

### Chiral Organic–Inorganic Hybrid Perovskites

**FIGURE 7:**
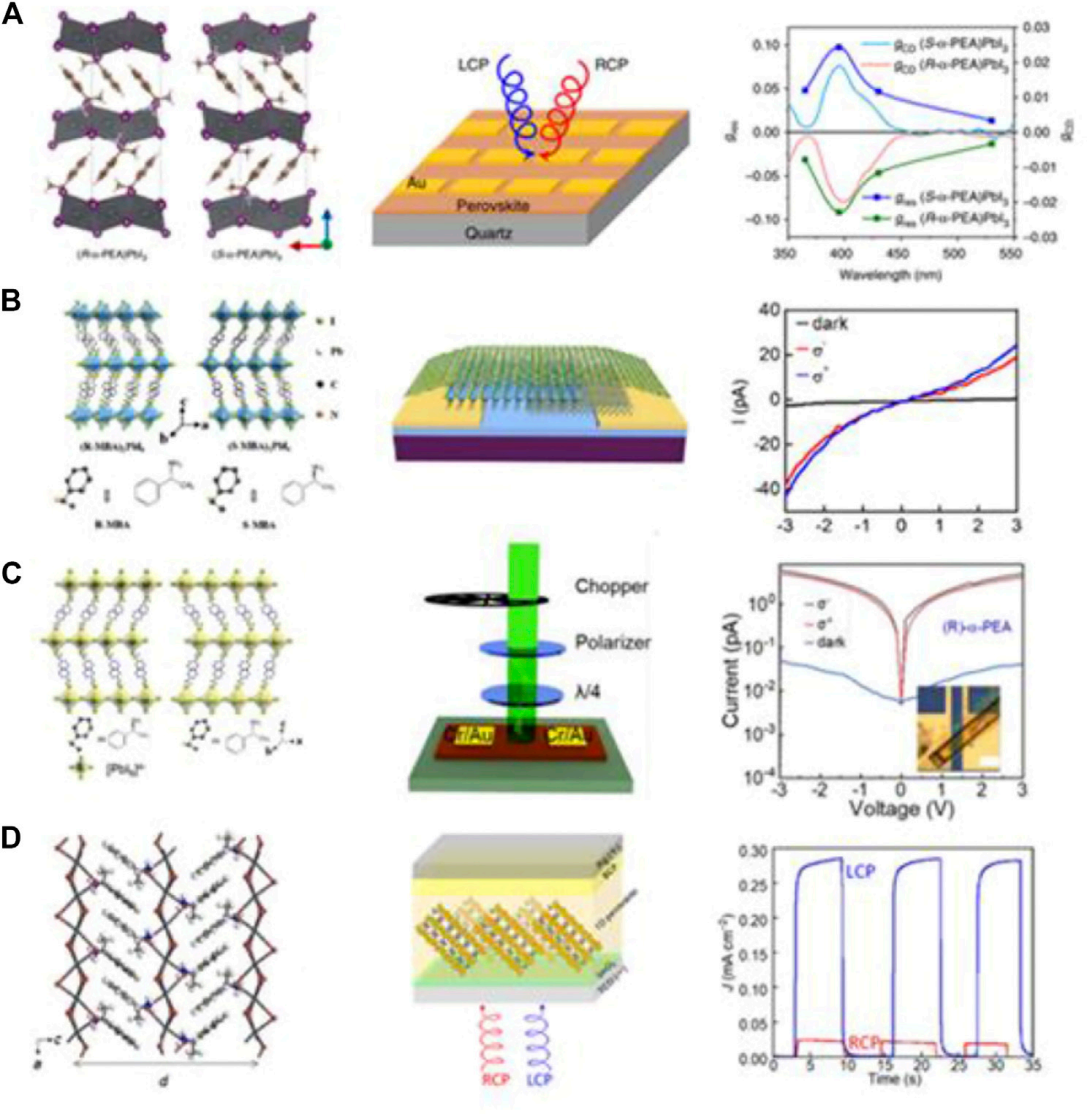
**(A)** Crystal structure of (*S*)-(α-PEA)_2_PbI_4_ and (*R*)-(α-PEA)PbI_3_. Schematic diagram of (*S*)-(α-PEA)PbI_4_ and (*R*)-(α-PEA)PbI_3_ film photodetectors. Wavelength-dependent g_res_ spectra of (*S*)- and (*R*)-(*α*-PEA)PbI_3_ thin film photodetectors. Reproduced with permission (Chen et al., 2019). Copyright 2018, Nature Publishing Group. **(B)** Schematic illustrations of the crystal structures of (*R*-and *S*-MBA)_2_PbI_4_, schematic illustration of the (R-MBA)_2_PbI_4_ microplate photodetector, and I–V curves of the hBN/(*R*-MBA)_2_PbI_4_/MoS_2_ heterojunction in the dark and under illumination with left-handed (σ) and right-handed (σ^+^) CPL. Reproduced with permission (Ma et al., 2019). Copyright 2020, American Chemical Society. **(C)** Schematic illustrations of the crystal structure of (*S*)-α-(PEA)_2_PbI_4_ and (*R*)-α-(PEA)_2_PbI_4_. Dark current and photocurrent of an (*R*)-α-(PEA)_2_PbI_4_ microplate two-probe device under illumination with 520 nm monochromatic light (Wang et al., 2019b). Reproduced with permission. Copyright 2020, America Chemical Society. **(D)** Structure model of (*R*-NEA)PbI_3_ in the ac plane. Schematic diagram of the helical 1D perovskite–based photodetector. Time course of the photoresponse (J–T curves) under monochromatic light (Ishii and Miyasaka, 2020). Copyright 2018, American Association for the Advancement of Science.

In recent years, organic–inorganic hybrid perovskites have been widely applied in optoelectronic applications because of their low-cost solution processing, tunable bandgap, high absorbance coefficient, strong defect tolerance, and solution processability (Katan et al., 2019; Lei et al., 2020; Wei et al., 2020). Their direct bandgap, high light absorption ability, and tunable absorption wavenumbers make perovskites promising candidates for optoelectronic applications (Dou et al., 2014; Lu et al., 2016; Wang and Kim, 2017; Zhenkun et al., 2018; Kwak et al., 2019; Xu et al., 2019; Lee et al., 2020). Furthermore, by combining the excellent optoelectronic properties with their chirality, chiral organic–inorganic hybrid perovskites may be promising active layers for use in CPL photodetectors (Bisoyi and Li, 2014; Ahn et al., 2017; Li et al., 2019). To prepare a CPL photodetector with excellent performance, the active layer of the device must simultaneously combine high CPL absorption and effective charge transfer. Organic molecules have intrinsic advantages for the absorption of CPL; on the contrary, since carriers are transported within the energy band for an inorganic compound, they generally show high carrier mobility compared with organic conjugated molecules. Thus, the combination of organic molecules and inorganic compounds may be an effective strategy for fabricating CPL photodetectors with excellent performance. Chiral halide perovskites can be synthesized by incorporating the chiral organic molecules into inorganic halide perovskite systems, and the resulting materials display effective carrier transport and handedness-sensitive optical absorption (Ma et al., 2019; Wang et al., 2019a). The first chiral organic–inorganic hybrid halide perovskite was reported by Billing and Lemmerer in 2003, who synthesized an organic–inorganic hybrid perovskite with only a single enantiomer (Billing and Lemmerer, 2006). Three years later, they reported the corresponding 2D chiral organic–inorganic hybrid perovskite (Billing and Lemmerer, 2006). Chiral organic–inorganic hybrid perovskites combine chirality with the excellent optical and electrical properties of perovskite materials, making them good candidates for CPL detection. In 2019, Tang’s group pioneered the construction of a CPL-sensitive photodetector based on chiral organic–inorganic hybrid perovskites (α-PEA)_2_PbI_4_ (Figure 7A). First, they introduced chiral α-phenylethylamine (α-PEA) into a halide perovskite to synthesize (α-PEA)_2_PbI_3_ organic–inorganic hybrid perovskites that combined the CPL-sensitive absorption induced by chiral organics and the efficient charge transport of inorganic frameworks (Chen et al., 2019). The device fabricated from these materials provided an effective method for direct CPL detection and achieved a responsivity of 797 mA W^−1^, a detectivity of 7.1 × 10^11^ Jones, a 3-dB frequency of 150 Hz for 395 nm CPL, and one-month stability. Combined, these are competitive features for CPL detection.

Two-dimensional (2D) Ruddlesden–Popper-type lead halide perovskites have recently been in the spotlight due to their better long-term stability compared with their 3D counterparts (Smith et al., 2014; Niu et al., 2015; Stoumpos et al., 2016; Wang et al., 2017). Furthermore, 2D perovskites are promising candidates for developing high-performance optoelectronic devices due to their highly tunable bandgaps, strong quantum confinement effects, and high optical absorption coefficients (Cao et al., 2015; Constantinos C. Stoumpos et al., 2016; Wei et al., 2020). Recently, Li et al. obtained strong chirality in pure 2D perovskites by incorporating the chiral organic ligands (*S*)-(+)-α-methylbenzylamine (*S*-MBA) and (*R*)-(+)-α-methylbenzylamine (*R*-MBA) into layered lead-iodide frameworks. The organic light-emitting diode (OLED) devices based on the chiral 2D perovskites (*R*-MBA)_2_PbI_4_ and (*S*-MBA)_2_PbI_4_ achieved an average degree of circularly polarized photoluminescence (PL) of 9.6 and 10.1% at 77 K, respectively. Moreover, CPL detection has been achieved in chiral 2D perovskite microplate/MoS_2_ heterostructured devices, which can efficiently differentiate between LCPL and RCPL. They display a responsivity of 0.45 A/W and a detectivity of 2.2×10^11^ Jones under 518 nm CPL illumination, as shown in Figure 7B. The high degree of the circularly PL and excellent CPL detection, together with the layered nature of pure chiral 2D perovskites, make them promising materials for developing spin-associated electronic devices based on 2D perovskites (Ma et al., 2019). Nevertheless, the synthesis of organic–inorganic hybrid perovskites often requires the use of toxic solvents, such as DMF/DMSO or heating, which increase the complexity of preparation. In 2019, Li et al. reported a new method to economically synthesize 1D and 2D perovskite single crystals with high crystalline quality and enhanced stability. Growth was accomplished by strictly controlling the pH of the solution. Both 1D and 2D single-crystal perovskites were synthesized using this method at ambient conditions, as shown in Figure 7C (Wang et al., 2019a). The OLEDs fabricated using (*S*)-(α-PEA)_2_PbI_4_ and (*R*)-(α-PEA)_2_PbI_4_ as the light emission layer produced circularly PL. The degrees of circularly polarized light emitted by (*S*)-(α-PEA)_2_PbI_4_ and (*R*)-(α-PEA)_2_PbI_4_ were 11.4 and 13.7%, respectively. Then, the chiral 2D perovskite was used as the active layer to construct an hBN/(*S*-MBA)_2_PbI_4_/MoS_2_-based circularly polarized photodetector, which showed different photocurrent values under the illumination of 518 nm left-handed CPL and right-handed CPL. The responsivity and the anisotropy of the device reached peak values of 0.6 A W^−1^ and 0.23, respectively. Furthermore, the device based on hBN/(*S*-MBA)_2_PbI_4_/MoS_2_ achieved a maximum specific detectivity (D*) of 3.06×10^11^ Jones and an external quantum efficiency (*EQE*) of 140% for σ^+^ illumination at 518 nm.

Although several groups have reported CPL photodetectors based on chiral organic–inorganic hybrid perovskites, there is a crucial issue in which the polarization discrimination of CPL photodetectors is low because of their intrinsically low CD signal, which restricts their practical applications for sensitive CPL detection. To solve this problem, Miyasaka et al. reported direct CPL detection by a photodiode using a helical one-dimensional (1D) structure with lead halide perovskites (*R*-NEA)PbI_3_ composed of naphthylethylamine-based chiral organic cations. The 1D structure with face-sharing (PbI_6_)^4-^ octahedral chains whose helicity is largely affected by chiral cations shows intense circular dichroism (CD) signals over 3,000 mdeg at 395 nm with a high anisotropy factor (*g*_*CD*_) of 0.04. This high CD enables photocurrent detection with effective discrimination between left-handed and right-handed CPL. The CPL detector based on these 1D perovskites achieved a maximum polarization discrimination ratio of 25.4 in the direct photocurrent-mode detection of CPL (Figure 7D). This material can be applied for full Stokes imaging in advanced optical devices (Miyasaka et al., 2020a).

In recent years, the chiral organic–inorganic hybrid perovskite–based CPL photodetectors have made significant progress with continuous performance enhancement. However, most of these reported chiral perovskites involve high concentrations of toxic Pb which will become the potential bottleneck for their further application. To solve this issue, Luo’s group developed two lead-free halide double perovskites, namely, [(R)-β-MPA]_4_AgBiI_8_ [(R)-β-MPA = (R)-(+)-β-methylphenethylammonium,1-R] and [(S)-β-MPA]_4_AgBiI_8_ [(S)-β-MPA = (S)-(–)-β-methylphenethylammonium,1-S]. Circular dichroism measurements reveal that these perovskites exhibit significant chirality induced by organic cations to distinguish the polarization states of CPL photons. They demonstrate unique chiral polar photovoltaic properties, and the resultant self-powered CPL detection is achieved without an external power source (unprecedentedly). Furthermore, an anisotropy factor up to 0.3 is obtained for the self-powered CPL detection, reaching the highest value among reported chiral perovskites (Li et al., 2021). To further achieve the detection of Stokes parameters, Zhao et al. developed Stokes-parameter photodetectors based on single-crystalline chiral-perovskite nanowire arrays. By integrating the intrinsic structural chirality of perovskites with the anisotropic dielectric function of nanowires, the photoresponse to the circularly and linearly polarized light was realized on a single material. The single-crystalline nanowires exhibit a high responsivity of 47.1 A W^−1^ and a specific detectivity of 1.24 × 10^13^ Jones. This work provides an effective strategy for the detection of Stokes parameters by using chiral perovskites (Zhao et al., 2021).

## Exploration of Circularly Polarized Light Detectors With Excellent Performance

Although CPL photodetectors have made significant progress in the past few years (Table 1), there are still several important issues to be addressed before they can be implemented in practical applications. For example, the performance of CPL detectors is far behind that of common photodetectors. Therefore, several strategies have been used to improve the performance of CPL photodetectors, including cholesteric liquid crystals, heterostructures, and doping.

**TABLE 1:** Comparison of the key parameters of the presented CPL photodetectors.

Chiral materials	Wavelength (nm)	*p*	*R* (A W^−1^)	D* (Jones)	*EQE* (%)	*g*_*res*_	Ref.
1-Aza[6]helicene	365	—	0.01	—	—	—	Yang et al. (2013a)
ProSQ-C6:PCBM	545	—	0.054	3.3×10^14^	22	—	Schulz et al. (2019)
ortho-π-Extended PDI double-[7]heterohelicene	730	2.45	0.45	2.1×10^10^	89	0.057	Zhang et al. (2021)
Single-crystalline CPDI-Ph NWs	460	7.57×10^4^	334	—	8.81×10^4^	—	Shang et al. (2017)
Hydrazine-doped C1CPDI-Ph-CF crystal	495	1122	1.71×10^7^	2.1×10^16^	3.0×10^5^	0.12	Shang et al. (2020)
P3CT/BN hybrid film	375	—	—	—	—	0.1	Kim et al. (2019)
Achiral P6BT induced by CPL	450	40.34	1.86 × 10^–5^	7.44×10^7^	1.62 × 10^–3^	1.94	Cheng et al. (2020a)
Plasmonic nanostructures	1340	—	39.3	9.9×10^14^	1.96×10^4^	-	Li et al. (2015)
Center-symmetrical “Y”-shaped gold antenna	1550	—	0.021	—	—	—	Xiao et al. (2019)
(α-PEA)_2_PbI_3_ perovskites	395	—	0.797	7.1×10^11^	—	0.1	Chen et al. (2019a)
Chiral 2D (MBA)_2_PbI_4_ perovskite	518	—	0.45	2.2×10^11^	—	—	Ma et al. (2019)
1D and 2D (α-PEA)_2_PbI_4_ perovskites	520	-	0.6	3.06×10^11^	140	0.23	Wang et al. (2019b)
Chiral lead-free hybrid perovskites	520	—	2.2 × 10^–5^	1.2×10^7^	—	0.3	Li et al. (2021)
Chiral-perovskite nanowire arrays	505	—	47.1	1.24×10^13^	—	0.15	Zhao et al. (2021)
Chiral cellulose nanocrystal films	405	—	—	—	—	—	Grey et al. (2018)
Carbon dot nanomaterials	450	—	—	—	—	—	Zheng et al. (2018)
Cholesteric liquid crystal films	830	—	300	—	4.5×10^4^	1.9	Han et al. (2020)

### Performance Optimization of Circularly Polarized Light Detectors Based on Cholesteric Liquid Crystals

**FIGURE 8:**
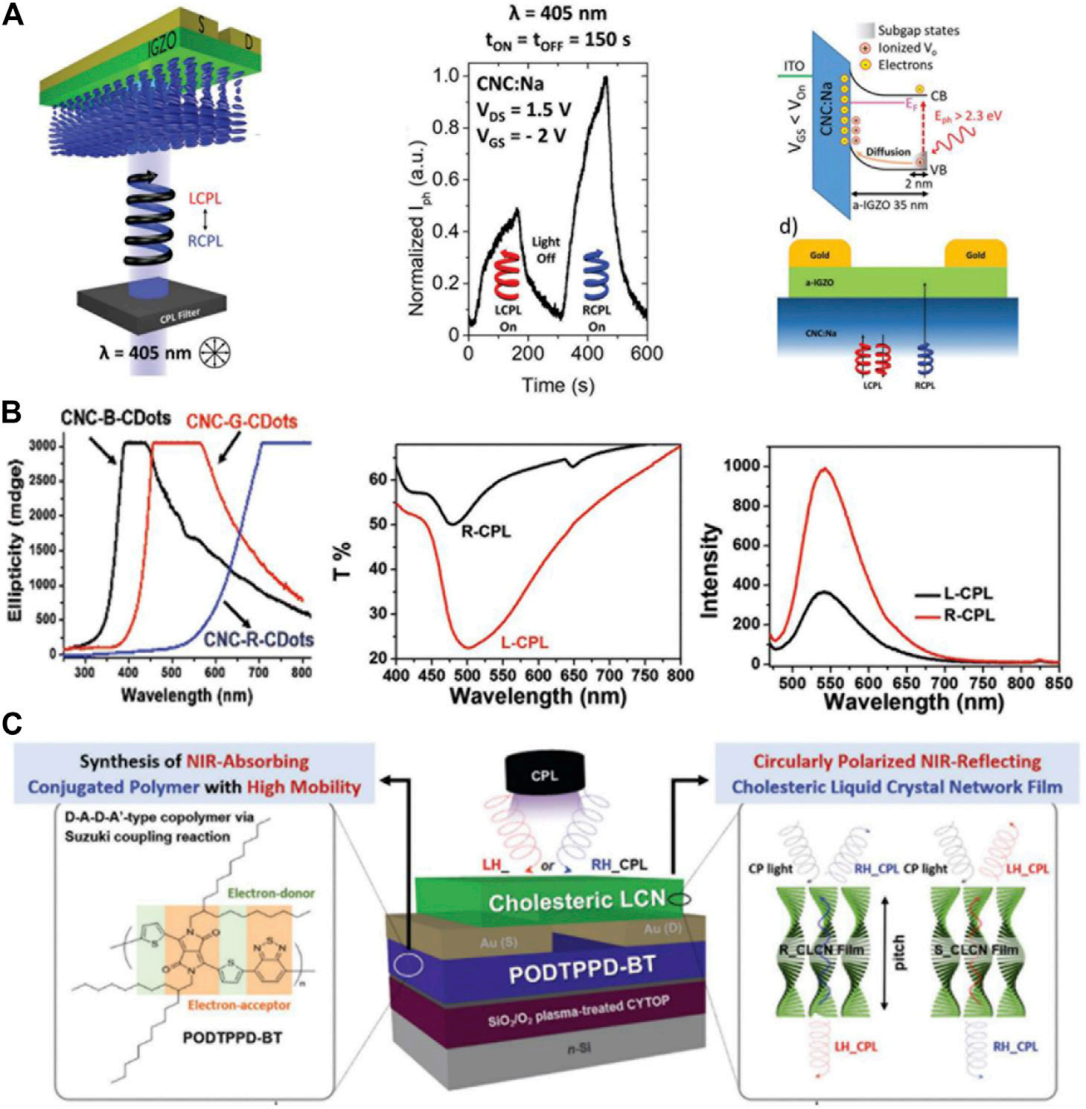
**(A)** A Schematic representation of the implementation of the CPL detection setup, normalized photogenerated current for 150 s LCPL, 150 s off, and 150 s RCPL cycles, and band diagram of ITO/CNC:Na/a-IGZO (Grey et al., 2018). Reproduced with permission. Copyright 2018, Wiley-VCH. **(B)** CD spectra of CNC-B-C dot films, CNC-G-C dot films, and CNC-R-C dot films. Transmission spectra of a CNC-G-C dot-483 film upon irradiation with LCPL and RCPL and photoemission spectra of a CNC-G-C dot-483 film (Zheng et al., 2018). Reproduced with permission. Copyright 2018, Wiley-VCH. **(C)** Schematic of the device structure of NIR CPL-OPTRs under circularly polarized NIR illumination and chemical structure of PODTPPD-BT (Han et al., 2020). Reproduced with permission. Copyright 2020, Wiley-VCH.

Cholesteric liquid crystals (CLCs) are ordered liquid crystal structures that exhibit intrinsic periodicity in the form of helical supramolecular structures. They have attracted special attention as optical diffraction gratings for light-emitting diodes, reflection-free lasers, tunable color filters, and reflective devices (San Jose et al., 2014; Ryabchun and Bobrovsky, 2018). Cholesteric structures provide an effective strategy to improve the performance of CPL photodetectors because of their various advantages. For example, they often show strong chiroptical selectivity, which allows them to reflect CPL with the same chirality and transmit CPL with the opposite chirality (Stranks et al., 2015). In addition, cholesteric liquid crystal network films prepared by cross-linking cholesteric liquid crystal molecules with helical supramolecular structures are easily integrated into devices (Kim et al., 2016). For example, cellulose nanocrystals are biocompatible, renewable, and low-cost nanomaterials. They can intrinsically self-assemble to form a left-handed chiral nematic ordered structure that is preserved in thin films (Shopsowitz et al., 2010; Klemm et al., 2011; Chu et al., 2015). Taking advantage of this property, cellulose nanocrystals also are suitable candidates for CPL detection. In 2018, Pereira et al. (Grey et al., 2018) used chiral cellulose nanocrystal films as gate dielectrics and amorphous indium-gallium-zinc-oxide (a-IGZO) as the semiconductor layer to fabricate a bottom-gate architecture transistor, as shown in Figure 8A. Experimental results revealed that around 35% LCPL was reflected by the CNC:Na layer, while around 90% of the RCPL was transmitted which reached the a-IGZO layer. Thus, the phototransistor showed a greater photocurrent when irradiatted by RCPL compared with LCPL because the CNC-G-C dot films showed differential absorption of RCPL and LCPL. Therefore, the device could be used to distinguish the two polarization states according to the different changes in photocurrent during CPL illumination. Furthermore, the obtained device achieved excellent performance with an on/off ratio of up to 7 orders of magnitude, with subthreshold swings around 80 mV dec^−1^ and saturation mobilities up to 9 cm^2^ V^−1^s^−1^. Carbon dots are remarkable inorganic phosphors with distinct features such as a high emission intensity, biocompatibility, and easy availability. These properties are superior to those of many inorganic quantum dots, making them promising materials for optoelectronic applications (Schlesinger et al., 2015; Tian et al., 2017; Han M. et al., 2018; Liu Y. et al., 2019; Sivasankarapillai et al., 2020). For example, Zheng et al. (2018) designed and fabricated a series of circularly polarized luminescent carbon dot nanomaterials. The freestanding films exhibited superior CPL strength, precise handedness, and tunable wavelengths from the near-ultraviolet to the near-infrared region by using different carbon dots and changing the photonic bandgap. The lower fluorescence quantum yield at the superimposed photonic bandgap and photoemission band provided compelling evidence for the photonic bandgap effect for stimulating CPL. They also demonstrated that the CPL strength depended on intrinsic factors such as photoemission intensity, carbon dot loading, and irradiation wavelength with respect to the photonic bandgap. More importantly, the predominant left-handed helical organization and the broad wavelength tunability of the photonic bandgap were harnessed for the detection of CPL, as shown in Figure 8B. This sentence has been modified to By introducing a cholesteric liquid crystal network films (CLCN) on the phototransistor based on small-bandgap conjugated polymer poly[{2,5-bis-(2-octyldodecyl)-3,6-bis-(thien-2-yl)pyrrolo[3,4-c]pyrrole-1,4-diyl}-co-{2,2′-(2,1,3-benzothiadiazole)]5,5′-diyl}] (PODTPPD-BT) as a reflector, Han et al. Han et al. (2020) reported a near-infrared circularly polarized light–sensing photodetector (NIR CPL-OPTR). Due to the ability of the CLC film to distinguish the polarization direction of CP light, the final NIR CPL-OPTR device achieved a dissymmetry factor of 1.9 (Figure 8C). Furthermore, the maximum photoresponsivity and external quantum efficiency reached 300 A W^−1^ and 4.5 × 10^4^, respectively. This work provides an effective strategy for preparing CPL photodetectors with high performance in the near-infrared region. In summary, the introduction of cholesteric liquid crystals in CPL photodetectors is an effective strategy for improving their performance due to the strong chiral selectivity of CLCs.

### Performance Optimization of Circularly Polarized Light Detector Based on Heterojunction

Photodiodes and phototransistors are the most widely used device structures for CPL detection. Determining how to improve the performance of these two devices is the key to the development of CPL detectors. Material systems and device structures can be improved to overcome the limitations of some fundamental aspects of CPL detectors. Heterojunctions may provide an effective strategy for improving the performance of CPL photodetectors because photoexcited charge carriers can be spatially separated at the hetero-interface, which can improve the separation efficiency of electron–hole pairs (Xu et al., 2013; Han J. et al., 2018; Liu H. et al., 2019; Wang et al., 2019b; Wu et al., 2019). For example, Kim et al. (2019) demonstrated a photodiode through blending P3CT with BN, and they showed that the annealing temperature and the BN content in the hybrid film greatly influenced the phase separation and crystallization of hybrid films. They found when the content of BN was 33 wt%, the hybrid film displayed the optimal composition for chiroptical sensing. Furthermore, a structure with a bilayer film of multifaceted BN microcrystals at the top surface and the P3CT/BN mixed layer at the bottom was obtained by thermal annealing at the optimal temperature (120°C). Subsequently, a CPL photodiode was fabricated by using a P3CT/BN(*R*) heterojunction thin layer as the active layer, and the CPL photodetector showed a higher photocurrent when illuminated by RCP, compared with LCP, which demonstrates its ability to distinguish left-handed and right-handed CPL. Moreover, the average dissymmetry factor of the photocurrent (g_Iph_) of the CPL detector reached 0.1, which is comparable to the values of previously reported CP photodiodes with excellent performance.

### Performance Optimization of Circularly Polarized Light Photodetectors Based on Doping

**FIGURE 9:**
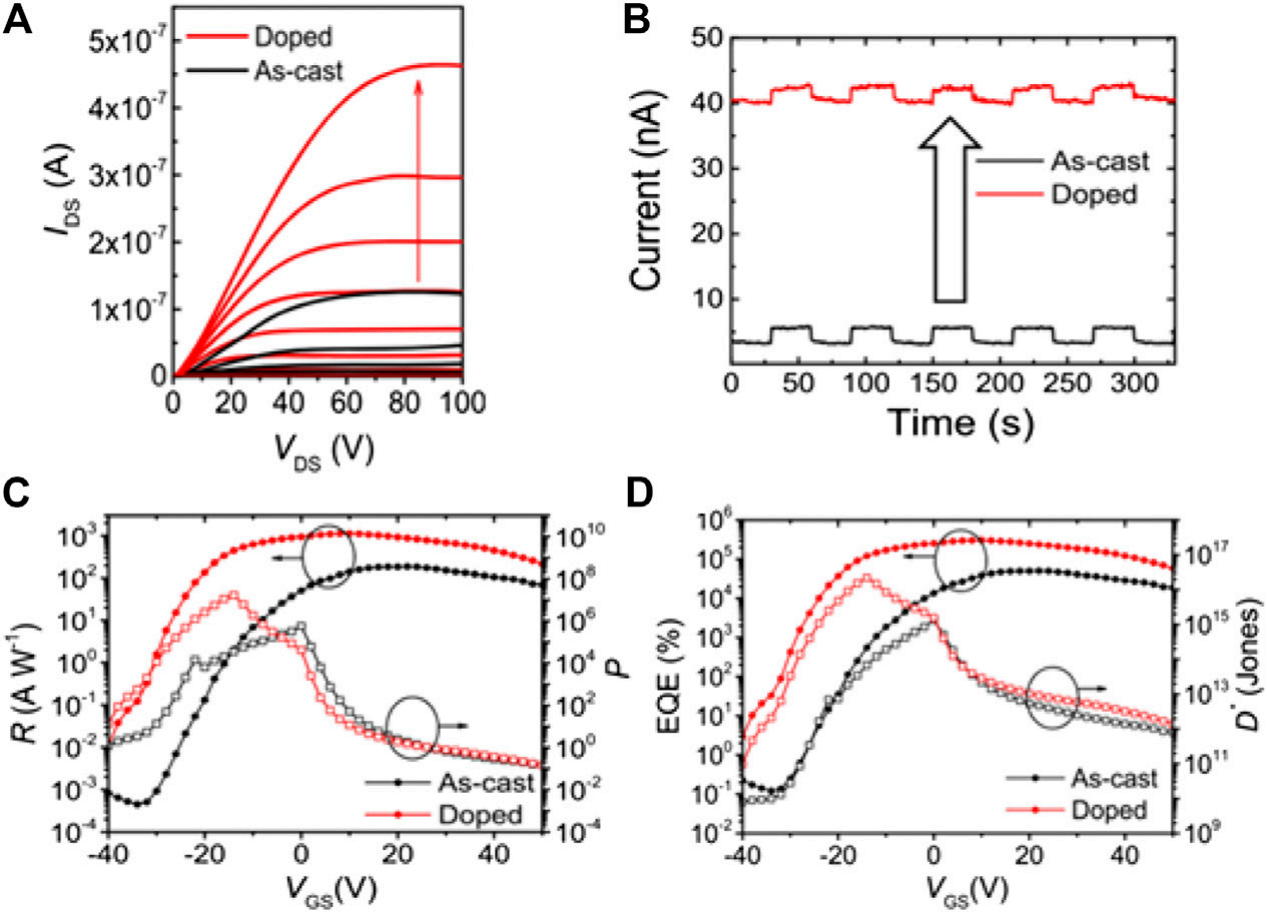
**(A)** Output characteristics of an (*R*)-CLCPDI-Ph-CF single crystal before and after hydrazine doping, **(B)** photo-switching curve of as-cast (undoped) and doped (*R*)-CLCPDI-Ph-CF single-crystal OPTs, **(C)** photoresponsivity (*R*) and photosensitivity (*p*), and **(D)** *EQE* and detectivity (D*) of OPTs based on an (*R*)-C1CPDI-Ph-CF single crystal and hydrazine-doped (*R*)-C1CPDI-Ph-CF single crystal, respectively. Reproduced with permission (Shang et al., 2020). Copyright 2020, American Chemical Society.

Several groups have reported that molecular doping using either strong electron donors or acceptors can improve the optoelectronics properties of organic semiconductors, mainly by tuning their Femi level (Chen Y. et al., 2019; Zhang et al., 2019). In addition, compared with bulk doping, surface doping is more appealing due to its less detrimental effect on organic semiconductors. For example, Shang et al. demonstrated a CPL phototransistor based on single crystals of (*R*)-C1CPDI-Ph-CF and (*R*)-C1CPDI-DMF. The OPT devices based on the (*R*)-C1CPDI-CF single crystal exhibited maximum *p*, *R*, D*, and *EQE* values of 1.7×10^7^, 1129 A W^−1^, 2.2×10^16^ Jones, and 3.0 × 10^5^%, respectively. Subsequently, they used hydrazine to dope the (*R*)-C1CPDI-CF single crystals, and there was an obvious increase in current after hydrazine surface doping (Figures 9A,B). Furthermore, the OPTs based on doped (*R*)-C1CPDI-CF single crystals showed improved *p* (∼30×higher), *R* (∼6×higher), D* (∼16×higher), and *EQE* (∼6×higher) values compared with undoped (*R*)-C1CPDI-CF single crystals as shown in Figures 9C,D. Density functional theory (DFT) analysis revealed that the enhanced performance of doped SC-OPTs was because of the increased electron affinity after absorption of hydrazine (Shang et al., 2020).

## Circularly Polarized Light Photodetector Mechanism

**FIGURE 10:**
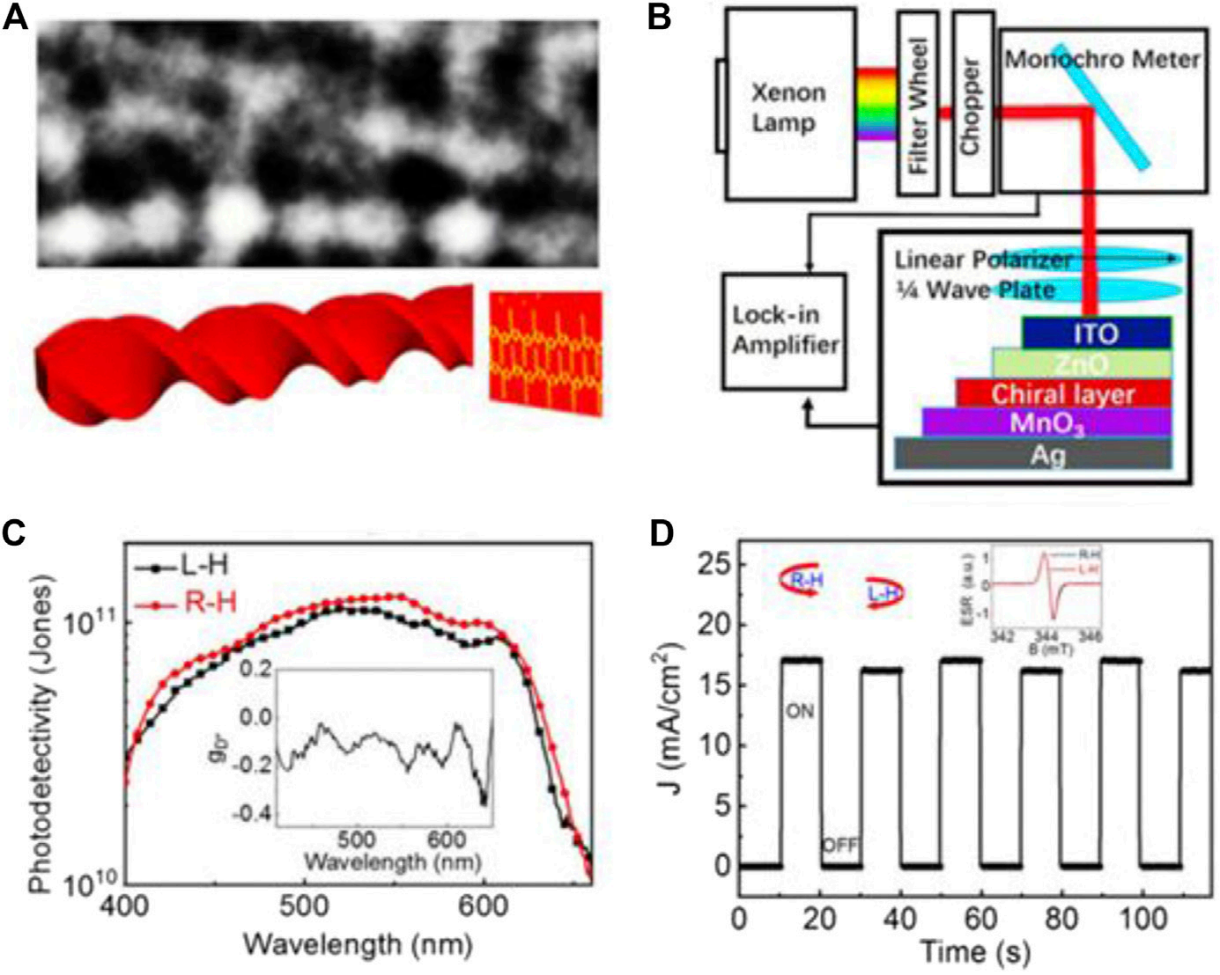
**(A)** TEM and scheme of the chiral PT nanowire structure, **(B)** schematic diagram of the setup used for CPL detection, **(C)** wavelength-dependent detectivity (D*) of the chiral PT–based photodetectors, and **(D)** photocurrent of ITO/ZnO/chiral PT:PCBM/MoO_3_/Ag under the stimuli of left-handed and right-handed CPL illumination (Wang et al., 2020). Reproduced with permission. Copyright 2020, American Institute of Physics.

Currently, the detection mechanism of CPL by circularly polarized light detectors based on chiral materials is not clearly understood, but it mainly includes 1) differential absorption of left-handed and right-handed CPL and 2) orbital angular momentum (OAM) generated by chirality. To elucidate the detection mechanism of CPL, Qin et al. reported a CPL photodetector based on chiral polythiophene (PT) induced by (*R*)-(+)-limonene. Figure 10A shows the TEM and scheme of the chiral PT nanowire structure. The device based on a chiral PT nanowire (Figure 10B) showed greater photocurrent and responsivity when irradiated with right-handed CPL compared with left-handed CPL as shown in Figure 10D, indicating that the device could effectively detect and distinguish left-handed and right-handed CPL. Moreover, the detectivity value (D*) of the CPL detector based on chiral polythiophene exceeded 1.6 × 10^10^ Jones ranging from 400 to 670 nm (Figure 10C), and it reached 1.25 × 10^11^ Jones at 553 nm. Most importantly, the experimental results showed that the differential absorption between LCPL and RCPL had a small contribution to the detection of CPL, while chirality-induced OAM played an important role in the CPL detection for achiral PT nanowire–based CPL photodetectors (Wang et al., 2020). This study plays a very important role in understanding the detection mechanism of CPL photodetectors.

## Potential Applications of Circularly Polarized Photodetectors

Due to their excellent optoelectronic properties, solution processability, compatibility with flexible substrates, and room-temperature operation, CPL photodetectors are promising candidates for practical optoelectronic applications, such as wearable electronics, polarization imaging, and secure communication (Wang et al., 2020; Han et al., 2020; Miyasaka, 2020b; Shang et al., 2020).

### Circularly Polarized Light Detectors for Wearable Optoelectronic Applications

**FIGURE 11:**
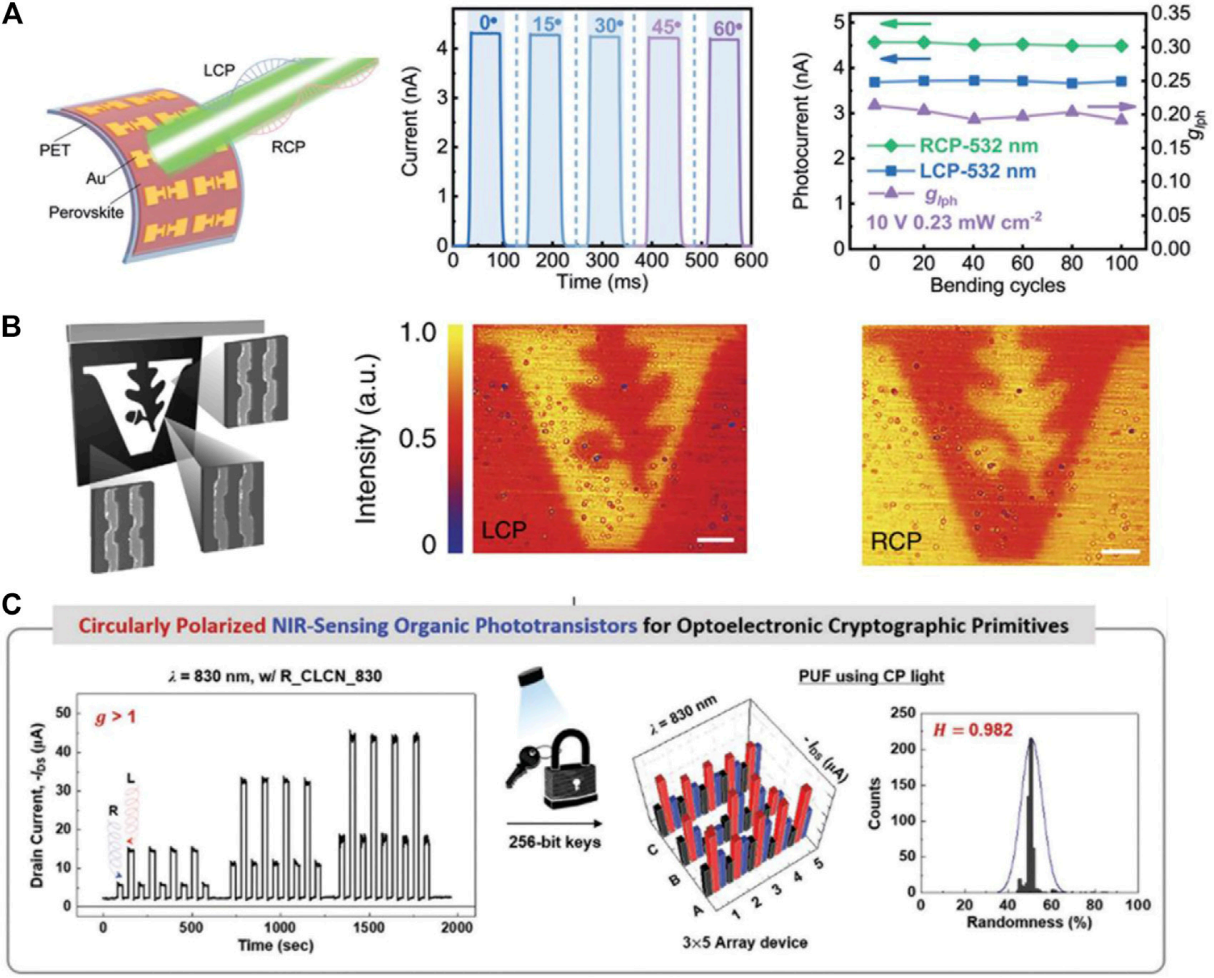
**(A)** Schematic illustration of a flexible CPL-sensitive photodetector based on the [(R)-βMPA]_2_MAPb_2_I_7_ film, I–t curves at different bending angles (0˚–60°), and photocurrent and *g*_*lph*_ as a function of bending cycles (Wang et al., 2020). Reproduced with permission. Copyright 2020, Wiley-VCH. **(B)** Schematic of the pattern with the LH metamaterial filling the black region and the RH metamaterial filling the white region. Photos of the metamaterial under LCP **(left)** and RCP **(right)** illumination (Li et al., 2015). Reproduced with permission. Copyright 2020, Nature Publishing Group. **(C)** Circularly polarized NIR–sensing organic phototransistors for optoelectronic cryptographic primitives (Han et al., 2020). Reproduced with permission. Copyright 2020, Wiley-VCH.

Wearable electronic devices are becoming increasing popular with consumers because they are lightweight, portable, and convenient and provide real-time monitoring (Gao et al., 2017; Kim et al., 2018; Xu et al., 2018). At the same time, CPL photodetectors based on chiral organic semiconductor materials and organic–inorganic hybrid perovskite materials can be fabricated on flexible substrates to form flexible CPL photodetectors, which show promising application prospects in the field of wearable electronics. For example, Wang et al. reported a flexible thin film [(*R*)-β-MPA]_2_MAPb_2_I_7_ photoconductor device on a PET substrate, as shown in Figure 11A. The flexible photodetector showed different photocurrent under illumination by 0.23 mWcm^−2^ RCP and LCP at 532 nm, demonstrating good CPL distinguishing ability (Wang et al., 2020). Furthermore, the flexibility of the CPL photodetectors was examined using bending testing at different curvatures, and the results showed that the corresponding photocurrents of a flexible device remain nearly unchanged at differed curvature radii, indicating that the flexible CPL photodetector showed excellent device robustness. Moreover, after 100 repeated bending/straightening cycles, the photocurrent and anisotropic factor of the flexible CPL photodetector changed by less than 10%. These results indicated that the flexible thin film CPL photodetector based on quasi-2D perovskites possesses superior mechanical flexibility and durability and represents a promising candidate for wearable optoelectronic applications.

### Circularly Polarized Light Detectors for Polarimetric Imaging

Since it provides abundant optical information, CPL has better imaging abilities than ordinary light. Even in poor weather conditions, CPL has good imaging ability, which cannot be realized using ordinary light imaging. Therefore, imaging based on CPL has more extensive application prospects for polarimetric imaging. For example, the application of CPL photodetectors in optical imaging has been reported by Valentine et al. (Li et al., 2015). They demonstrated an ultracompact CPL detector that could distinguish LCP and RCP due to the structure of the antenna layer, rather than the material’s intrinsic chirality. Furthermore, a spatially non-uniform, pixelated photodetector was fabricated by placing LH and RH chiral metamaterials into a single 90 × 90 μm array, containing about 100,621 unit cells. The Vanderbilt University logo was created by implementing the array with both LH and RH enantiomers, where the LH and RH chiral metamaterials filled the black and the white space of the logo, respectively. The letter “V” did not appear under illumination by linearly polarized or non-polarized light, while it showed clear contrast under illumination by LCP and RCP, as shown in Figure 11B. This study shows that CPL photodetectors hold great promise for polarization imaging.

### Circularly Polarized Light Photodetectors for Secure Communication Applications

Apart from the potential application prospects in the fields of imaging and wearable optoelectronics, CPL photodetectors also have application prospects in the field of secure communication. For example, Lim et al. reported a high-performance NIR CPL photodetector by incorporating a cholesteric LC film on the phototransistor based on the conjugated polymer PODTPPD-BT. The optimized NIR-OPTRs achieved a maximum responsivity of 12 A W^−1^ when illuminated by unpolarized NIR light due to the high NIR absorption and excellent charge transport properties of the PODTPPD-BT thin film. Moreover, due to the CLC film’s ability to distinguish the polarization direction of CP light, the final device demonstrated a maximum dissymmetry factor of responsivity 1.9, as well as a high photoresponsivity of 300 A W^−1^. Furthermore, to explore the possibility of using NIR-OPTRs for secure communication, a 3 × 5 array was fabricated, as shown in Figure 11C. By using near-infrared CPL, the array was encoded to generate secret keys, which enhanced the cryptographic characteristics, indicating that the NIR-OPTR is a promising approach for highly secure cryptographic primitives (Han et al., 2020).

## Summary and Outlook

In this review, we summarized the progress of circular polarization detectors in the past few years. The material systems and device structures have been enriched and optimized. The active materials range from the chiral enantiomer 1-aza[6]helicene synthesized by complex methods in 2013 to chiral structure metamaterials in 2015 and to chiral organic–inorganic perovskite materials in 2019. Furthermore, the methods to prepare photosensitive CPL materials and the detector performance have been greatly improved. For instance, the sensing region of CPL detectors based on chiral nanostructured materials has been extended to 1340 nm, and the responsivity (*R*) has been improved to 300 A W^−1^. Furthermore, the anisotropy factor (*g*_*res*_) of CPL photodetectors has increased to 1.9, demonstrating excellent distinguishing ability between LCP and RCP photons. These improvements in device performance and preparation methods play a very important role in the practical applications of CPL photodetectors.

Although the research of CPL photodetectors had made significant progress in the past few years, this area is still in its infancy, and there are still several important issues that need to be addressed before they can be implemented in practical applications. First, a limited number of chiral materials that have been used as active layers can distinguish between LCPL and RCPL. Furthermore, the mobility of CPL detectors is still not sufficient, and the photoresponsivity of polarized light detectors is far behind that of unpolarized light detectors. The anisotropy factor of responsivity, which is used to evaluate the ability to differentiate between LCPL and RCPL of CPL detectors, is also not high enough. The development of new chiral materials with both high mobilities and large CD values should be the main target of this research area. New strategies for controlling the aggregation structures and crystallization properties of thin films must be further explored. In addition, it is important to develop new device structures and optimize device interfaces for improving the performance of CPL photodetectors. The practical applications of CPL photodetectors, such as quantum optics, remote sensing, security surveillance, and drug screening, will be challenging. With the development of more chiral materials with excellent electrical properties and larger CD values and the development of more mature device technologies, CPL photodetectors based on chiral materials will become increasingly important in optical detection research.

Considering the development of CPL photodetectors in the past few years, we speculate their potential avenues for development as follows:

1. There are still limited types of active layer materials available for CPL photodetectors, Hence, it is important to develop materials that can combine strong optical activity with excellent optoelectronic properties.
2. With the progress in technology, the electronic devices with more functions have become popular in the market. Therefore, multifunctional CPL photodetectors would pave a potential direction to move ahead.
3. Stability is one of the key issues in industrialization of CPL photodetectors based on chiral materials. Therefore, enhancement of stability of CPL photodetectors can be one of the future directions.

As described here, we firmly believe that the CPL photodetectors based on chiral materials will attract more interest in the future and become a “hot topic” of research in this field.
